# De novo assembly of the desert tree Haloxylon *ammodendron* (*C. A. Mey.*) based on RNA-Seq data provides insight into drought response, gene discovery and marker identification

**DOI:** 10.1186/1471-2164-15-1111

**Published:** 2014-12-15

**Authors:** Yan Long, Jingwen Zhang, Xinjie Tian, Shanshan Wu, Qiong Zhang, Jianping Zhang, Zhanhai Dang, Xin Wu Pei

**Affiliations:** Institute of Biotechnology, Chinese Academy of Agricultural Sciences, Beijing, 100081 China; Gansu Agricultural Academy, Crop Institute, Lanzhou, 730070 China

**Keywords:** *Haloxylon ammodendron*, Drought, Transcriptome, Digital gene expression, EST-SSR

## Abstract

**Background:**

*Haloxylon ammodendron* (*C. A. Mey.*) is widely distributed across a range of habitats, including gravel desert, clay desert, fixed and semi-fixed sand, and saline land in Asian and African deserts. To date, no genomic information or expressed sequence tag-simple sequence repeat (EST-SSR) marker has been reported for *H. ammodendron* plants.

**Results:**

Using Illumina sequencing technology, we generated over two billion bases of high-quality sequence data on *H. ammodendron* and conducted de novo assembly and annotation of genes without prior genome information. These reads were assembled into 79,918 unigenes (mean length = 728 bp). Based on similarity searches comparing these unigenes with known proteins in the non-redundant (nr) protein database, 25,619 unigenes were functionally annotated with a cut-off E-value of 10^-5^. In addition, DGE reads were mapped to the assembled transcriptome for gene expression analysis under drought stress. In total, 1,060 differentially expressed genes were identified. Among these genes, 356 genes were upregulated after drought treatment, and 704 genes were downregulated. We used the KEGG database to annotate these drought-induced genes; 207 unigenes were identified in the KEGG pathway annotation, and approximately 12.1% of the unigenes with known function fell into categories related to fatty acid metabolism, starch and sucrose metabolism, and nitrogen metabolism, suggesting that these pathways or processes may be involved in the drought response. Together, a total of 35 drought-inducible transcription factors were identified, including WRKY, MYB and bZIP family members.

**Conclusions:**

Our study is the first to provide a transcriptome sequence resource for *H. ammodendron* plants and to determine its digital gene expression profile under drought conditions using the assembled transcriptome data for reference. These data provide a valuable resource for genetic and genomic studies of desert plants under abiotic conditions.

**Electronic supplementary material:**

The online version of this article (doi:10.1186/1471-2164-15-1111) contains supplementary material, which is available to authorized users.

## Background

Drought is one of the most common environmental abiotic stresses in the world. To adapt to environmental changes, plants have a variety of physiological responses and defence systems for withstanding drought conditions. The regulatory mechanism in higher plants has been analysed by studying a number of genes responding to drought stress at the transcriptional level [1, 2]. In *Arabidopsis thaliana*, for example, thousands of genes are thought to be involved in abiotic stress [3]. In general, drought stress-inducible genes have been classified into two groups: one group that directly protects plants against environmental stresses, and a second group that regulates gene expression networks and signalling in stress responses, such as in response to drought or salt stress [2]. Recently, progress has been made in analysing the functions of stress-inducible genes, not only to understand the mechanisms of drought stress but also to improve the drought tolerance of plants by gene transfer. Genetic studies have identified many transcription factors that are extensively involved in the regulatory network of drought-inducible genes [2, 3], including, for example, the NAC [4], WRKY [5], and MYB families [6].

As a xerophytic desert tree, *Haloxylon ammodendron* has great drought and salt resistance; thus, it plays an important role in the maintenance of the structure and function of the entire ecosystem in which it grows. *H. ammodendron* reduces wind speed and ameliorates the forest microclimate, thereby facilitating the settlement and growth of other desert plants [7]. *H. ammodendron* is widely distributed in a variety of habitats, including gravel desert, clay desert, fixed and semi-fixed sand, and saline land in the Asian and African deserts [8]. In China, about 56% of *H. ammodendron* is found in Xinjiang province, 40% in Inner Mongolia province, and the remaining 4% in Qinghai and Gansu provinces [9]. At present, most research examining the drought tolerance of *H. ammodendron* focuses on physiology [10–12] and on the identification of specific genes. Some important drought-related genes have been cloned using RT-PCR and the RACE method, including the *CMO* [13], *ARF1* [14] and *EF-hand CaBP* [15] genes. However, to our knowledge, no study involving large-scale drought-related gene screening and EST-SSR identification has been published to date.

Genome-wide analyses have dramatically improved the efficiency of gene discovery. With the advent of next-generation sequencing, large-scale transcriptome data has become available in both model and non-model species. Since Hegedus et al. [16] first used Solexa/Illumina’s Digtal Gene Expression (DGE) system to study the zebrafish transcriptome after *Mycobacterium marinum* infection, RNA-Seq and DGE technology have been widely used to identify plant genes, including those expressed in stress condition [17, 18], related to important agronomic traits. For example, in *A. thaliana*, about 30% of the transcriptome was found to be regulated by abiotic stress, and 2,409 genes were identified as being of great importance in cold, salt, and drought tolerance [3]. In Chinese cabbage, Yu et al. (2012) conducted transcriptome profiling by tag sequencing and by quantifying the expression of more than 10,000 genes in response to dehydration stress; these authors found that 28 genes in 37 transcription factors were involved in signal transduction and that the expression of 61 water- and osmosensing-responsive genes was significantly altered in response to water deficit [19].

Molecular markers play important roles in many activities involved in plant breeding, such as studies of genetic diversity [20], marker-assisted selections [21], and the identification of genes responsible for desirable traits [22]. Molecular markers have been widely used to map important genes and to assist with the breeding of trees. Many EST-SSR markers were developed using collected ESTs or high-throughput sequencing data. For example, in the rubber tree, Li et al. [23] used Hiseq2000 sequencing to sequence RNA from the bark of healthy rubber trees and got more than 30 million sequencing reads; after these sequences were assembled, 22,756 unigenes were obtained. A total of 39,257 EST-SSRs were then identified from these 22,756 unigenes. Finally, the PCR success ratio for the 110 randomly selected primers used in this study was 96.36% [23]. In the date palm, Zhao et al. (2012) identified 4,609 EST-SSRs from 28,889 EST sequences, and after examining their random primers, these authors found that one third of their primers had polymorphisms in 12 different date palm cultivars [24].

In this study, the transcriptome from different tissues of drought-treated and control *H. ammodendron* plants were sequenced with Illumina paired-end sequencing technology. The resulting sequence data were assembled and annotated, DGE profiling was performed, and EST-SSR markers were developed. To our knowledge, this is the first systematic report on the transcriptome of *H. ammodendron*. This research is essential to understand the transcriptional changes underlying the drought response in *H. ammodendron*. The transcriptome data generated from our study provide a resource for gene annotation and discovery, the development of molecular markers, genomic and transcriptomic assembly, and the development of microarrays for *H. ammodendron*. In addition, the EST-SSR markers predicted and developed in this study enlarge the number of available molecular markers and may facilitate gene mapping, linkage map development, genetic diversity analysis, and marker-assisted selection breeding in *H. ammodendron*.

## Results

### Illumina paired-end sequencing and *de novo*assembly

To investigate the transcriptome of *H. ammodendron* under drought conditions, RNA was extracted from different tissues and sequenced using Illumina paired-end sequencing technology. In this study, a total of 74,011,190 raw sequencing reads with a length of 100 bp each were generated from a 200 bp insert library. After removing adaptors and low quality data, 70,947,290 clean reads were obtained. The high-quality reads were then used to assemble the transcriptome data using Trinity software. Using overlapping information in high-quality reads, a total of 153,589 transcripts were generated, with an average length of 1,060 bp and an N50 of 1,812 bp. After compared the different transcripts representing one unigene, the longest length transcript for each unigene was extracted. So then a total of 79,918 unigenes were obtained. The average length was 728bp, and transcripts with lengths of more than 500 bp accounted for about 37.15% of all transcripts (Table 1, Additional file 1).

**Table 1:** **Summary of the** ***Haloxylon ammodendron*** **transcriptome**

Category	Number				Total number	Mean length (bp)	N50 (bp)	Total nucleotides
	200-500 bp	500-1 kbp	1 k-2 kbp	>2 kbp				
Transcripts	65,913	29,962	33,638	24,076	153,589	1,060	1,812	162,880,991
Unigenes	50,226	13,322	9,722	6,648	79,918	728	1,345	58,206,305

### Annotation of all non-redundant unigenes

For the validation and annotation of the assembled unigenes, all assembled unigenes were screened against the NCBI non-redundant (nr) and SWISS-PROT protein databases using the BLAST 2.2.28+ program with an E-value threshold of 1e-5. Among 79,918 unigenes, 25,619 (32.05%) were found to have significant similarity to 20,988 unique proteins. Of all the unigenes, 18,272 (22.86%) with significant identities to SWISS-PROT proteins were matched with 11,269 unique protein accessions (Table 2). It was found that a smaller percentage was obtained when searching against the SWISS-PROT protein database rather than against the nr database. In total, BLAST searches identified 18,221 unique protein accessions from the nr and SWISS-PROT protein databases, suggesting that our Illumina paired-end sequencing had likely captured a substantial proportion of the drought-response genes in *H. ammodendron*.

**Table 2:** **Summary of the functional annotation of assembled unigenes**

Public database	Number of unigenes	Percentage (%)
Annotated in nr	25,619	32.05
Annotated in nt	11,402	14.26
Annotated in KO	7,246	9.06
Annotated in SWISS-PROT	18,272	22.86
Annotated in PFAM	20,469	25.61
Annotated in GO	22,586	28.26
Annotated in KOG	9,680	12.11
Annotated in all databases	3,338	4.17
Annotated in at least one database	29,989	37.52
Total Unigenes	79,918	100

### Functional classification by GO and COG

**Figure 1:**
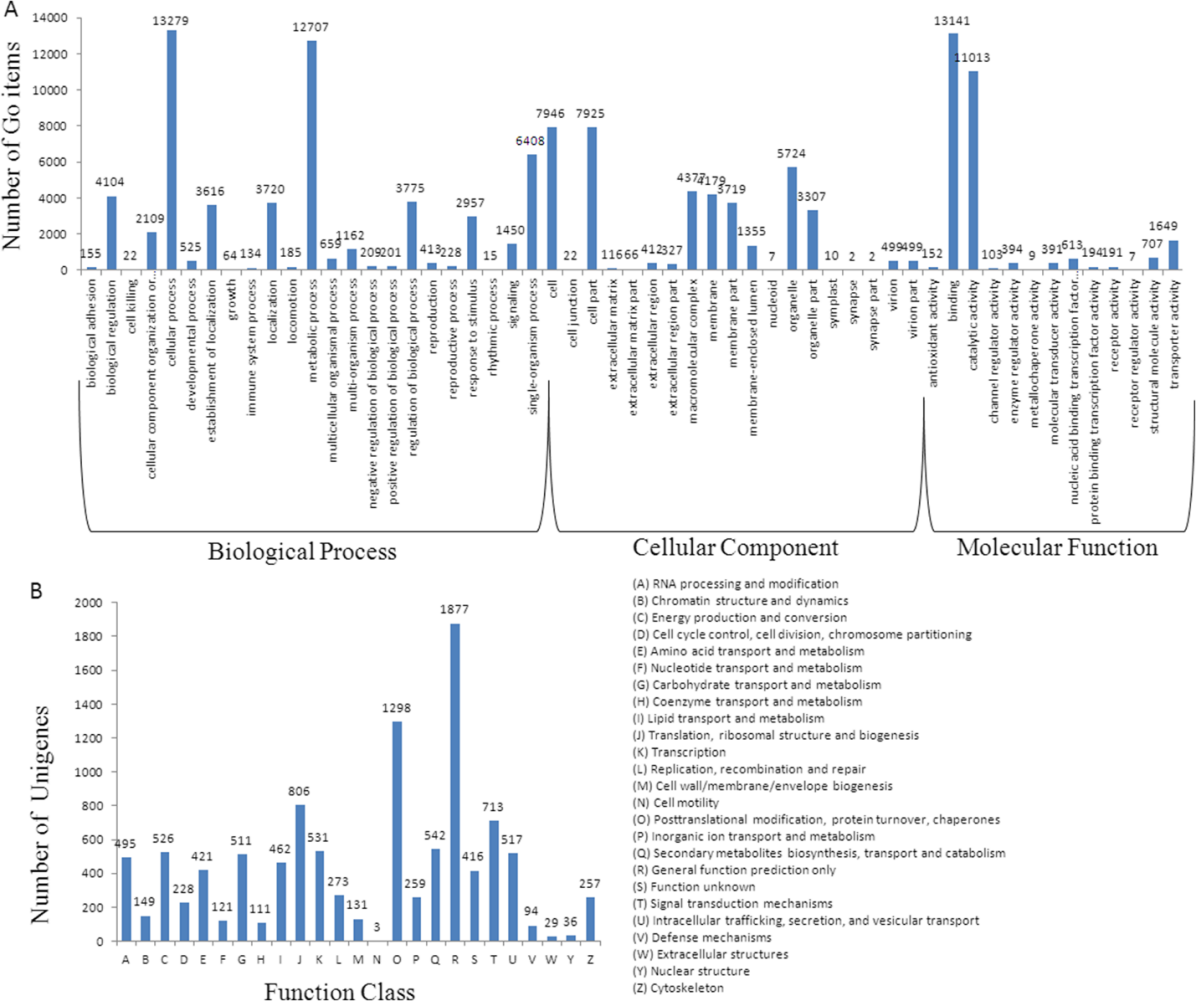
**Functional classification of the assembled unigenes. (A)** Functional classification of the assembled unigenes based on Gene Ontology (GO) categorisation. The results are summarised in three main GO categories: biological processes, cellular components and molecular functions. The x-axis indicates the subcategories, and the y-axis indicates the numbers related to the total number of GO terms present; the unigene numbers that are assigned the same GO terms are indicated at the top of the bars. **(B)** Histogram of Clusters of Orthologous Groups (COG) classification. The unigenes were aligned to the COG database to predict and classify possible functions. Out of 25,619 hits in the NCBI non-redundant (nr) database, 10,806 unigenes were annotated and separated into 25 clusters.

In order to classify the functions of the predicted *H. ammodendron* unigenes, Gene ontology (GO), which is an internationally standardised gene functional classification system, was used. In total, 22,586 unigenes with BLAST matches to known proteins were assigned to GO classes using 1,734 functional terms (Table 2, Additional file 2). As shown in Figure 1A, the majority of the unigenes were assigned to the categories of biological processes (58,097, 45.69%), followed by cellular components (6,085, 29.81%) and molecular functions (40,494, 24.5%). Under the category of biological processes, cellular processes (13,279, 22.86%) and metabolic processes (12,707, 21.87%) were prominently represented, indicating that important cellular processes and metabolic activities occurred in *H. ammodendron* in response to drought. Under the classification of molecular functions, binding (13,141, 46%) and catalytic activities (11,013, 38.5%) were separately the first and second largest categories, respectively, whereas other categories, such as those for transporter activities, enzyme regulator activities, molecular transducer activities, and others, together contained only 4,410 unigenes representing 15.44% of the total number of unigenes. As for the cellular component, two categories, pertaining to cells and cell parts, accounted for approximately 39.19% of the cellular components that were identified; the organelle category accounted for approximately 14.14% of the cellular component unigenes, and the membrane and membrane part categories accounted for 21.13%.

In order to predict and classify possible functions, all unigenes were aligned to the Cluster of Orthologous Groups (COG) database in which orthologous gene products were classified. Out of 25,619 unigenes with significant similarity to nr proteins in this study, 10,806 sequences were assigned to COG classifications (Figure 1B). Among the 25 COG categories, the cluster related to general function prediction (1,877, 17.37%) was the largest group, followed by those for posttranslational modification (1,298, 12.01%); translation, ribosomal structure and biogenesis (806, 7.46%); and signal transduction mechanisms (713, 6.6%).

### Functional classifications using KEGG pathways

**Figure 2:**
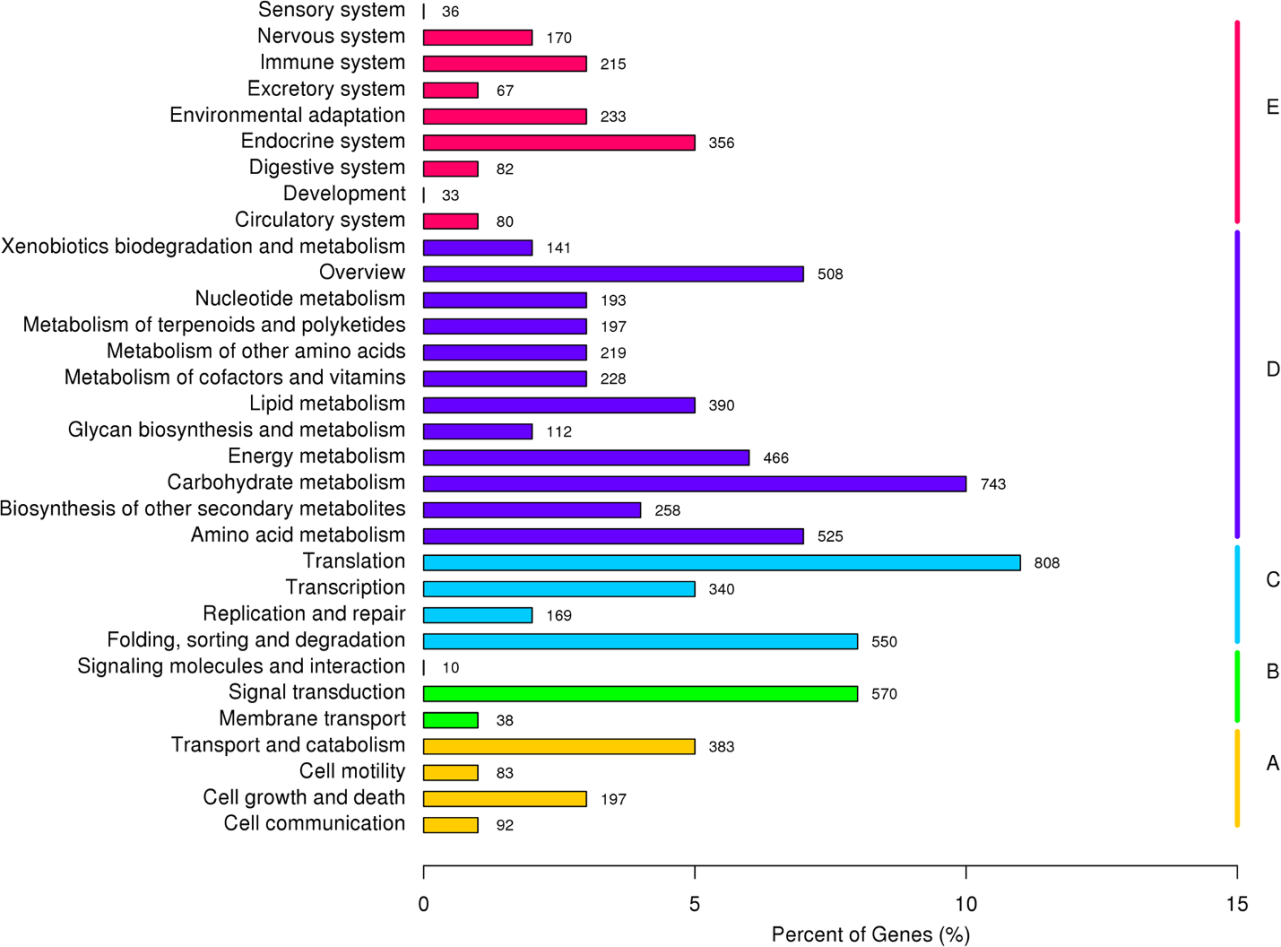
**Pathway assignment based on the Kyoto Encyclopedia of Genes and Genomes (KEGG) database. (A)** Classification based on cellular process categories, **(B)** classification based on environmental information processing categories, **(C)** classification based on genetic information processing categories, **(D)** classification based on metabolism categories, and **(E)** classification based on organismal systems categories.

The Kyoto Encyclopaedia of Genes and Genomes (KEGG) pathway database is a knowledge base for the systematic analysis of gene functions in terms of networks of genes and molecules in cells and their variants specific to particular organisms. To further analyse the transcriptome of *H. ammodendron*, all the unigenes were analysed with respect to the KEGG pathway database. Out of the 79,918 identified unigenes, 11,155 (13.96%) with significant matches in the database were assigned to 5 main categories that included 265 KEGG pathways (Figure 2, Additional file 3). Among the 5 main categories that were identified, metabolism was the category with the greatest number of unigenes (5,326, 47.75%), followed by genetic information (2,044, 18.39%), organismal systems (1,781, 16.02%), cellular processes (1022, 9.19%) and environmental information processing (982, 8.83%). These results indicate that active metabolic processes were occurring in the drought treatment condition. As shown in Additional file 3, the KEGG metabolism category contained 12 sub-categories, including carbohydrate metabolism, nucleotide metabolism, biosynthesis involved in secondary metabolism, amino acid metabolism, lipid metabolism, and energy metabolism, among others.

### Analysis of differential gene expression during the drought process

**Figure 3:**
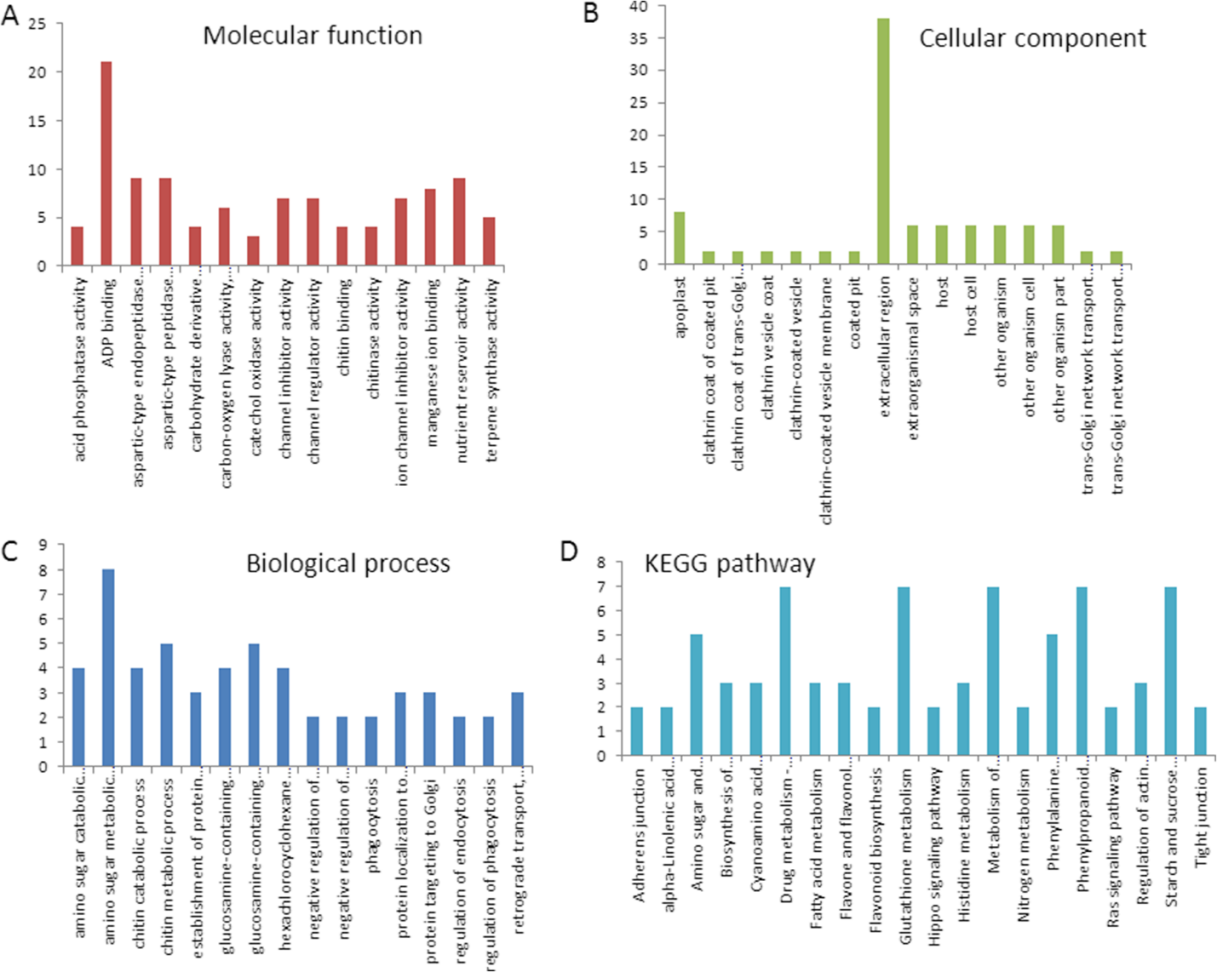
**Proportions of DEG unigenes categorised by comparison with the GO and KEGG databases. (A)** Functional classifications based on GO in the molecular function category; **(B)** functional classifications based on GO in the cellular component category; **(C)** functional classifications based on GO in the biological process category; and **(D)** functional classifications based on KEGG pathways. The y-axis indicates the number of unigenes in a given main category. The x-axis indicates the specific sub-categories of unigenes in that main category.

To reveal the molecular events occurring during the drought process, two digital gene expression (DGE) libraries were constructed using RNA from the two pools of control and drought treatment RNA samples and sequenced using Illumina technology. After Illumina sequencing and the removal of adaptors and bad-quality reads, approximately 11,019,037 and 12,958,245 reads were obtained for the two control replicates, and 13,915,918 and 12,960,189 reads were obtain for the two replicates for the drought-treated plants. We then mapped the clean reads to the transcriptome reference data, and a total of 57,695 and 55,998 unigene sequences were identified for the control replicates, and 58,788, and 57,596 unigene sequences were identified for the drought replicates. After calculating the expression level of each mapped unigene, a total of 1,060 unigenes were detected that had levels of expression that were significantly different between the drought-treated and control libraries. No homologue was found in the NCBI database for 469 differentially expressed unigenes. Both upregulation and downregulation of unigene expression occurred among the differentially expressed unigenes. Among all differentially expressed unigenes, 356 were induced by drought treatment, and 704 were downregulated after one week of drought treatment. However, only 261 unigenes were functionally annotated with GO terms (Figure 3A-C), and only 207 unigenes were identified in the KEGG pathway database (Figure 3D). Interestingly, approximately 12.1% of the unigenes with known function fell into the categories related to fatty acid metabolism, starch and sucrose metabolism, and nitrogen metabolism (Figure 3D), suggesting that these pathways and processes may participate in the drought response.

Previous studies have shown that transcription factors have a major effect on the network of drought-response genes. In this study, a total of 35 unigenes encoding known or putative transcription factors were found, including the WRKY, MYB, and ethylene-responsive transcription factors (Additional file 4).

### Expression of selected genes differentially regulated between the two DGE libraries

**Figure 4:**
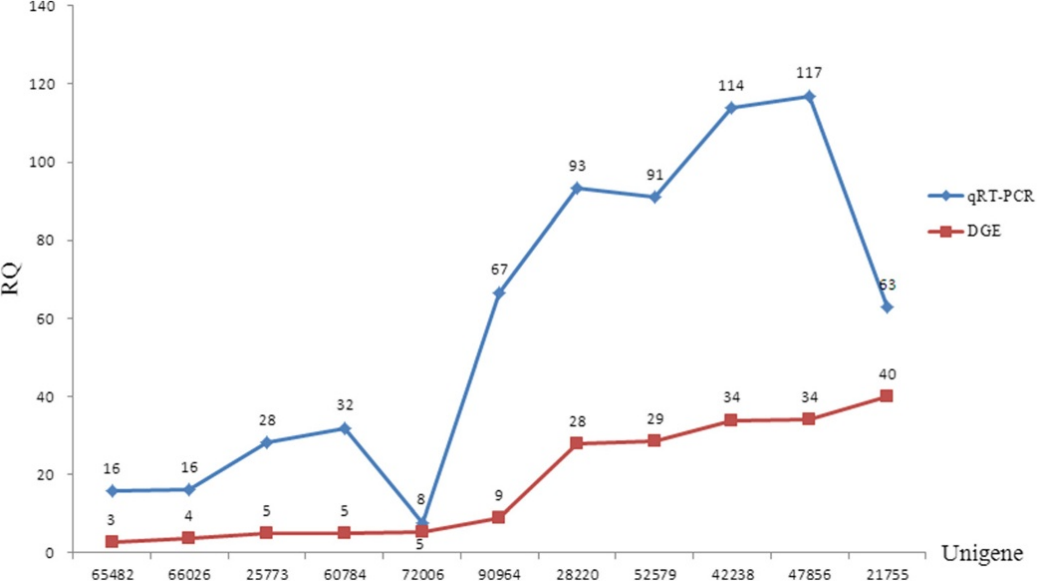
**Unigene expression tendencies in both DGE sequencing data and qRT-PCR experimental results.** The x-axis shows the different unigenes, and the y-axis represents the drought quantity relative to control levels. The numbers shown above the two graphs indicate the fold changes for each unigene for the drought treatment relative to control conditions.

To confirm the gene expression data, 18 unigenes whose expression was upregulated in drought plants were randomly chosen from the two libraries for qRT-PCR analysis. Among them, 11 unigenes were significantly upregulated in drought-treated plants, while the expression levels of the remaining 7 unigenes were not significantly different between the two libraries. As shown in Figure 4, the unigene expression trends were similar in both DGE sequencing and qRT-PCR data. After examining the gene annotation information available for these unigenes, it was found that information on gene function was available for three unigenes; however, no matches in any database were obtained for the remaining 8 unigenes. These results suggest that some drought-resistance pathway genes may work together to defend the plant from drought stress.

### Development and characterisation of EST-SSR markers

To further evaluate the quality of the sequence data assembly and to develop new molecular markers, the 79,918 unigenes generated in this study were used to mine potential microsatellites that were defined as mono- to hexanucleotide motifs with a minimum of three repetitions. Using the MISA software (http://pgrc.ipk-gatersleben.de/misa/misa.html), a total of 17,310 potential simple sequence repeats (SSRs) were identified in 13,840 unigenes. Of these 13,840 unigenes, 11,078 and 2,762 unigenes contained one or more than one SSR, respectively (Table 3). The number of potential EST-SSRs per unigene varied from 1 to 8, with an average of 1.25.

**Table 3:** **Summary of the EST-SSRs that were identified in the transcriptome**

Search item	Number
Total number of examined unigenes	79,918
Total size of examined sequences (bp)	58,206,305
Total number of identified EST-SSRs	17,310
Number of EST-SSRs containing sequences	13,840
Number of sequences containing more than one EST-SSR	2101
Mononucleotide	10,630
Dinucleotide	2316
Trinucleotide	3969
Tetranucleotide	351
Pentanucleotide	23
Hexanucleotide	21

**Figure 5:**
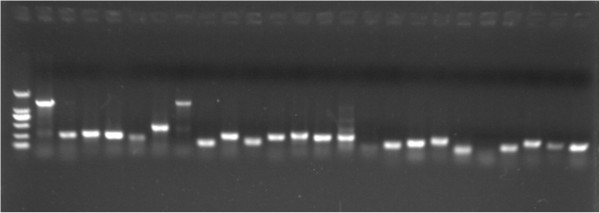
**Photograph of PCR amplification results for the EST-SSR markers in** ***H. ammodendron.*** The first line is the DNA ladder. The subsequent lines are the PCR products generated using different primers.

Using the SSR-containing sequences, 113 SSR sites were randomly selected to design EST-SSR primers with the Primer Premier 3.0 software. Information about these EST-SSR primers is shown in Additional file 5. Among these 113 primer pairs, 96 were used successfully to PCR-amplify genomic DNA (Figure 5), while the remaining seventeen pairs of primers failed to generate PCR products at several annealing temperatures. Among the PCR products of the 96 working primer pairs, 87 PCR products appeared to result from specific amplification; among these 87 PCR products, 78 PCR products were of the expected sizes, while the other six PCR products were larger than the expected sizes, suggesting that the amplified regions likely contained introns. Nine primer sets yielded PCR products that separated into more than one band, which may have resulted from either the primer design or the high heterozygosity of the *H. ammodendron* germplasm.

## Discussion

In this study, a large number of *H. ammodendron* transcriptomic unigenes (79,918) were sequenced using the Illumina HiSeq 2000 platform (Table 1). The N50 length of the unigenes was 1,345 bp, and the average length was 728 bp; these results were comparable to those obtained in recently published transcriptomic analyses of other plant species, such as *Reaumuria soongorica* (N50 = 1,109 bp, average length = 677 bp [25]) and *Litchi* (N50 = 811 bp, average length = 601 bp [26]). To date, Trinity is one of most powerful software packages used for the de novo assembly of short reads. In this study, fewer than half of the unigenes (29,989, 37.52%) identified were successfully annotated using BLAST searches of the public nr, nt, SWISS-PROT, GO, COG and KEGG databases, given the absence of genomic information on *H. ammodendron* (Table 2). Notably, the percentage of unigenes that were annotated is the lowest among previous studies conducted using the same sequencing strategy during the previous year (55 to 78.9%, [25, 27–29]). It is possible that a larger percentage could not be annotated in this study due to technical limitations, such as sequencing depth or read length [30], that are common to all studies that perform de novo transcriptome analyses. We found that the unannotated sequences were, on average, much shorter than the annotated unigenes (402 bp vs. 975 bp).

The C4 pathway has been acknowledged to be more adaptive than the C3 pathway in response to abiotic stresses, such as high temperature, radiation and drought [31].

*H. ammodendron* is a C4 plant, according to its physiological characteristics [31]. In this study, most of the genes encoding key enzymes involved in the C4 carbon fixation pathway were presented in the transcriptomic dataset from the annotation of the KEGG pathway. The gene expression data confirmed the C4 character of *H. ammodendron*. The 17 C4 pathway genes that were identified were all downregulated in drought-treated plants compared with the controls. This result is consistent with the previous finding that the efficiency of the photosystem decreases in conditions of water deficiency.

It has long been known that extensive changes in gene expression occur when plants are exposed to drought stress [1]. Generally, both upregulation and downregulation of gene expression occur under drought conditions. It has been reported that more genes are upregulated than are downregulated under drought stress in model plants [32]. In *A. thaliana*, 16,744 genes have been found to be drought responsive; it is interesting to note that after 2 h of treatment, 1,188 (7%) were found to be upregulated and 217 (1.3%) downregulated, while after 10 h of treatment, nearly the same percentage of genes (12.3%) was upregulated as was downregulated [3]. In cotton, over 16% of the genome exhibited altered expression levels in response to drought stress. Among the genes whose expression levels were altered, 5,344 genes were induced by drought shock, and 4,630 were downregulated with 2 days of treatment [33]. In the present study, 1,060 differentially expressed genes were identified, but the proportion of the genome that is devoted to drought stress is unknown due to a lack of genome resources for this species. Approximately half of the genes examined were found to be drought-inducible. These results suggest that plants vary in their abilities to adapt to drought stress. Gene expression in desert plants may differ extensively from that in inland plants, and the differences in desert plants’ gene expression patterns may confer an enhanced ability to respond to drought stimuli. Among the differentially expressed genes regulated by drought stress in *H. ammodendron*, over 50% had no homologues in the NCBI database. Some of these genes may represent novel drought-responsive transcripts that have not been reported in other plants.

EST-SSR markers are very important for research on a variety of topics, including the assessment of genetic diversity, the development of genetic maps, comparative genomics, marker assisted selection breeding, and others. To our knowledge, no previous study has reported the identification of ESR-SSRs in this desert tree. Transcriptome sequencing provided a large number of sequences that could be used to develop EST-SSR markers in the *H. ammodendron* tree. In total, 17,310 potential EST-SSRs were identified in 13,840 unigenes. In this study, in addition to the more common dinucleotide, trinucleotide and other nucleotide repeats that were included in the selection, mononucleotide repeat SSRs were also included, and the proportion of EST-SSRs that were mononucleotide repeats was larger than those of the other types of repeats. Trinucleotide repeats were the next-most abundant type, followed by dinucleotide repeats, consistent with previous reports [34]. The most abundant dinucleotide and trinucleotide motifs were AG/TC and AAG/TTC, respectively. These results are consistent with previous results for dicots such as oak trees [35] and castor bean plants [36]. Of 113 primer pairs randomly selected for PCR validation, 96 (85%) produced clear bands. The PCR success rate was similar to that observed in several previous studies, such as in a study of poplar trees [34], but higher than that reported in a study by Triwitayakorn et al. (75%) [37]. Therefore, the 17,310 potential EST-SSRs identified in this study will provide a wealth of resources for developing EST-SSRs in the desert tree.

## Conclusions

In this study, we used high-throughput sequencing data to characterise the transcriptome of *H. ammodendron*, a species for which few genomic data are available. DGE sequences were mapped to the assembled transcriptome for further gene expression analysis. A large number of candidate genes involved in drought stress were identified. Furthermore, a set of EST-SSRs were identified that have specific PCR products. This data represents a fully characterised transcriptome and provides a valuable resource for genetic and genomic studies in desert plants.

## Methods

### Sample collection and preparation

*H. ammodendron* seeds were provided by the Gansu Desert Control Institute. The seeds were sowed on damp filter papers and incubated at 4°C for 4 days before being placed at 23°C under long-day (16 h light/8 h dark) conditions with a photosynthetic photon flux density of 150μmol m^-2^ s^-1^. The seedlings were grown in four pots (20 seedlings/pot) representing two replicates of two treatments. After the seedlings grew for one month, one set of seedlings were treated with a one-week (7d) stress, and the second set of seedlings was used as a control and received no treatment. The control pots were irrigated from the bottom every day, while drought-stressed pots were not irrigated and were monitored for wilting symptoms. We checked the drought phenotype when treated without watering, and found that the seedlings were wilting for one week not watering. So we chose this condition for finding the genes response to drought stress. Several tissues, including leaves, stems and roots, were then harvested from the drought and control samples for subsequent RNA isolation.

### RNA isolation and transcriptome sequencing

Total RNA was extracted from the two replicates of the drought and control plants with TRIzol Reagent (Invitrogen, 15596–026) according to the manufacturer’s instructions. The four RNA samples that were of sufficient quality were used to construct the transcriptome sequence library. The total four RNA from each sample was then pooled to one, using equivalent quantities of each sample. The transcriptome sequencing library was generated using NEBNext Ultra RNA Library Prep Kits for Illumina(NEB, USA) following manufacturer’s instructions. Following the instructions provided by Illumina, mRNA was purified from the pooled, total RNA using polyT oligo-attached magnetic beads (Novogene, China). Fragmentation buffer was added to disrupt the mRNA into short fragments. Reverse transcriptase and random primers were used to synthesise the first strand cDNA from the cleaved mRNA fragments. The second strand cDNA was synthesised using buffer, dNTPs, RNase H, and DNA polymerase I. The double strand cDNA was purified using QIAquick PCR extraction kits (QIAGEN, Hilden, Germany) and washed with EB buffer for end repair and single nucleotide A (adenine) addition. Finally, sequencing adaptors were ligated onto the fragments. The required fragments were purified by AMPure XP beads and enriched by PCR to construct a library for transcriptome sequencing.

### Data filtering and de novo sequence assembly

The transcriptome library was sequenced using the Illumina HiSeq 2000 system. The sequencing-received raw image data were transformed by base calling into raw sequence data, which were termed raw reads. The raw data were then filtered by data-processing steps to generate clean data via a process that included the removal of adapter sequences, reads in which unknown bases are greater than 10%, and low-quality sequences (in which the percentage of low-quality bases of quality value ≤ 5 is greater than 50% in a read). After the clean data was generated, transcriptome assembly was accomplished using Trinity software [38] with min_kmer_cov set to 2 by default and all other parameters set to default values. The raw data are available in the Gene Expression Omnibus (GEO; http://www.ncbi.nlm.nih.gov/projects/geo/) under accession number GSE63970.

### Functional annotation of unigenes

For functional annotation, the assembled unigenes that might putatively encode proteins were searched against the nr (http://www.ncbi.nlm.nih.gov/), SWISS-PROT (http://www.expasy.ch/sprot/), KEGG (http://www.genome.jp/kegg/) and COG (http://www.ncbi.nlm.nih.gov/cog/) databases using the BLASTX algorithm. A typical cut-off value of E-value < 1e-5 was used. With Nr annotations, the Blast2GO program [39] was used to assign GO annotations to the unigenes according to component function, biological process and cellular component ontologies. After getting GO annotations for all unigenes, WEGO software [40] was used to assign GO functional classifications to all the unigenes and to understand the distribution of gene functions for the species on the macro level.

### DGE library preparation, sequencing and mapping analysis

Total RNA from different tissues, including leaves, stems, and roots, were extracted from drought-treated and control plants for two replicates. Each DGE library included pooled RNA from 4 plants. A total of 3 μg RNA per sample was used as input material for the RNA sample preparations. The procedure for constructing the DGE sequencing libraries was the same as that for constructing the transcriptome sequencing libraries. After the raw data was generated and the data-processing steps were completed, the clean reads were then mapped to the assembly transcriptome reference sequences using RSEM software [41]. Mismatches of no more than 2 bases were allowed in the alignments. The read count for each gene was obtained from the mapping results. The DGE data are available in the Gene Expression Omnibus (GEO; http://www.ncbi.nlm.nih.gov/projects/geo/) under accession number GSE63970.

### Identification of differentially expressed unigenes

Gene expression levels were calculated based on the numbers of reads mapped to the reference sequence, using the FPKM [42] method. After calculating gene expression levels, the differentially expressed genes (DEGs) were screened by comparing gene expression levels. Implementing the method described by Anders [43], differential expression analysis of two conditions was performed using the DESeq R package (1.10.1). DESeq provides statistical routines for determining differential expression in digital gene expression data using a model based on the negative binomial distribution. The resulting P values were adjusted using Benjamini and Hochberg’s approach for controlling the false discovery rate. In this study, unigenes with an adjusted P < 0.1 found by DESeq were considered differentially expressed.

### Quantitative real-time PCR validation

To confirm the DGE results, quantitative real-time reverse transcription PCR (qRT-PCR) was performed. Eighteen unigenes were randomly chosen for qRT-PCR analysis in the two libraries. The primers employed in the qRT-PCR experiments are listed in Additional file 6. qRT-PCR was implemented using the SYBR premix Ex Taq kit (TaKaRa, Dalian, China) on an ABI 7500 Real-Time System (Applied Biosystems), with the first strand cDNA serving as the template. Eighteen transcripts were randomly chosen for qRT-PCR analysis. The assembled *Actin* unigene (comp15413_C0) was used as an internal control. The relative quantitative method (△△CT) was used to calculate the fold change in the expression levels of target genes [44]. All reactions were performed in three technical replicates using one biological sample.

### Development and detection of EST-SSR markers

The MISA software (http://pgrc.ipk-gatersleben.de/misa/misa.html) was used to identify microsatellites in the unigenes. In this study, EST-SSRs were considered to contain motifs consisting of one to six nucleotides. Primers for each SSR were designed using Primer3 software (http://primer3.ut.ee). In total, 113 pairs of primers were designed (Additional file 5) and validated by PCR analysis. The DNA for PCR amplification was extracted from the control samples using the CTAB method [45]. PCR amplification was conducted as follows: PCR mixtures were held at 94°C for 4 min, followed by 35–40 cycles of 94°C for 30 s, 55-60°C for 30 s and 72°C for 30s. The final extension was performed at 72°C for 10 min. The PCR products were analysed by electrophoresis on 1.0% agarose gels.

## Electronic supplementary material

Additional file 1: **Length distributions of assembly transcripts and unigenes.** (TIFF 35 KB)

Additional file 2: **Summary of the GO classifications of assembled unigenes.** (XLSX 808 KB)

Additional file 3: **Summary of the KEGG classifications of assembled unigenes.** (XLSX 37 KB)

Additional file 4: **Transcription factors identified among the DEGs.** (XLSX 17 KB)

Additional file 5: **Primer information for EST-SSRs.** (XLSX 16 KB)

Additional file 6: **Primer information for qRT-PCR analysis.** (XLSX 10 KB)
