# Supplementary figures and images for: De novo assembly of the desert tree Haloxylon ammodendron (C. A. Mey.) based on RNA-Seq data provides insight into drought response, gene discovery and marker identification
Source: BMC Genomics. 2014 Dec 15;15(1):1111. doi: 10.1186/1471-2164-15-1111 (PMC4377846; doi:10.1186/1471-2164-15-1111)

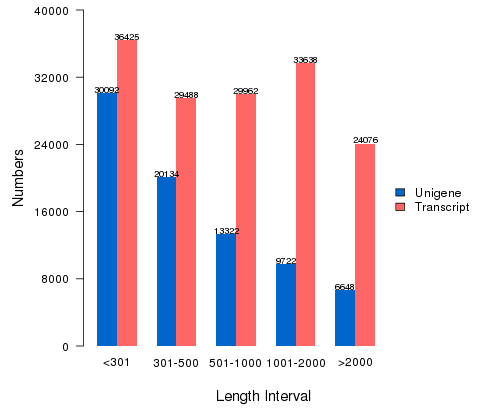

Supplement: Supplementary file 1 — Additional file 1: Length distributions of assembly transcripts and unigenes. (TIFF 35 KB) [file 12864_2014_6835_MOESM1_ESM.tiff]
